# Assessing the Impact of Differential Genotyping Errors on Rare Variant Tests of Association

**DOI:** 10.1371/journal.pone.0056626

**Published:** 2013-03-05

**Authors:** Morgan Mayer-Jochimsen, Shannon Fast, Nathan L. Tintle

**Affiliations:** 1 Department of Mathematics, Scripps College, Claremont, California, United States of America; 2 Department of Operations Research, Massachussets Institute of Technology, Boston, Massachusetts, United States of America; 3 Department of Mathematics, Statistics and Computer Science, Dordt College, Sioux Center, Iowa, United States of America; University of California, Irvine, United States of America

## Abstract

Genotyping errors are well-known to impact the power and type I error rate in single marker tests of association. Genotyping errors that happen according to the same process in cases and controls are known as non-differential genotyping errors, whereas genotyping errors that occur with different processes in the cases and controls are known as differential genotype errors. For single marker tests, non-differential genotyping errors reduce power, while differential genotyping errors increase the type I error rate. However, little is known about the behavior of the new generation of rare variant tests of association in the presence of genotyping errors. In this manuscript we use a comprehensive simulation study to explore the effects of numerous factors on the type I error rate of rare variant tests of association in the presence of differential genotyping error. We find that increased sample size, decreased minor allele frequency, and an increased number of single nucleotide variants (SNVs) included in the test all increase the type I error rate in the presence of differential genotyping errors. We also find that the greater the relative difference in case-control genotyping error rates the larger the type I error rate. Lastly, as is the case for single marker tests, genotyping errors classifying the common homozygote as the heterozygote inflate the type I error rate significantly more than errors classifying the heterozygote as the common homozygote. In general, our findings are in line with results from single marker tests. To ensure that type I error inflation does not occur when analyzing next-generation sequencing data careful consideration of study design (e.g. use of randomization), caution in meta-analysis and using publicly available controls, and the use of standard quality control metrics is critical.

## Introduction

In anticipation of a tidal wave of next-generation sequencing data from large case-control studies, numerous statistical tests intended to boost statistical power have been proposed. These tests attempt to aggregate genotype-phenotype association across numerous single nucleotide variant sites in a region of interest [1]–[11]. This new class of “rare variant tests” is beginning to be applied to real sequence data, as well to both imputed and genotype array data. However, aside from *in silico* simulation studies comparing the methods, relatively little is known about the behavior of these methods on real sequence data.

One of the first, large-scale attempts to understand rare variant tests when applied to real sequence data was at Genetic Analysis Workshop 17 where analyses revealed that existing rare variant tests perform poorly on real sequence data: with both increased type I errors and low statistical power [12]. Numerous potential explanations for the poor performance have been suggested including population stratification, gametic phase disequilibrium between causal and non-causal variants and genotyping errors–both differential and non-differential [13]–[16]. Recently, other simulation [17] and mathematical [Liu et al., unpublished manuscript] analyses have attempted to better understand the behavior of these tests.

Addressing population stratification, gametic phase disequilibrium and other related issues likely amount to analytic challenges which will be solved as methods for the analysis of sequence data mature. However, genotyping errors, long known to impact power and type I error in single marker (common variant) tests of genotype-phenotype association, have typically been robust to analytic advances, unless alternative study designs are employed [18]–[20]. Thus, it is useful to consider the impact of genotyping errors on current rare variant tests of association.

For single marker tests, when genotyping errors occur according to an error process that is unrelated to the phenotype (non-differential genotype errors), power loss is observed [21]–[26]. Recently, we considered the impact of non-differential genotype errors on rare variant tests of association [27]. We found that even at very low genotype error rates, misclassifying common homozygotes as heterozygotes translates into substantial power loss for rare variant tests, an effect that is magnified as the minor allele frequency (MAF) at the site decreases. Additionally, we demonstrated that at low error rates, heterozygote to homozygote errors have little impact on power. Heterozygote to (common) homozygote errors can be common in practice, and, at moderate to large error rates, can also substantially impact power.

Differential errors, genotyping errors that result from a process that is different for cases and controls, are well-known to inflate the type I error for single marker tests of association [28], [29]. Specifically, Ahn et al. [29] found that type I errors increase as MAF decreases, as differential errors coding more common to less common genotypes increase, and as sample size increases. However, no work has been done to explore the impact of differential genotyping errors on the new class of rare variant tests of association. Since low MAF markers are the most prone to type I errors from differential genotyping errors and rare variant tests are, by their very nature, focused on the rarest of variants, it is especially prudent to explore the impact of differential error on rare variant tests.

There are numerous plausible explanations for differential error processes in rare variant data. As is the case for single-marker tests of association, without good study designs which ensures random assignment of samples to sequencing centers, to individuals handling the samples, to sequencing machines, etc., genotyping errors can easily occur at different rates in the cases and controls. One particular area of concern is the increasing trend to use publicly available databases of controls. When using publicly available databases, there is no random assignment of cases and controls to sequencing pipelines, thus there are numerous ways that differential genotyping errors can be introduced. Furthermore, even if publicly available datasets are simply being used to impute rare variants, the potential for differential genotyping imputation error exists. Similarly, when using a Bayesian prior based on the known MAF at the variant site to call rare genotypes there is a potential for differential genotyping errors when the Bayesian prior favors variants observed more frequently in the cases or controls.

In this manuscript, we conduct a comprehensive simulation study to evaluate the extent to which differential genotyping errors impact the type I error rate of five recently proposed rare variant tests of association. We also evaluate the factors associated with increased type I error rate in rare variant tests of association.

## Methods

### Simulation of Genotypes and Phenotypes

The methods used to simulate genotypes in this study have been described elsewhere [27].We provide a brief overview here. We considered a total of four different genotype distributions at the locus of interest. Namely, all possible combinations of the following two parameters: Low/High number of single nucleotide variants (SNVs; 8 or 64 at the locus) and Low/High MAF of the SNVs at the locus (0.1%/1% or 0.5%/5%). At each locus ¾ of the SNVs are more common (1% or 5% MAF), and ¼ are less common (0.1% or 0.5%). Genotypes were simulated independently and assuming Hardy-Weinberg Equilibrium. Sample sizes (sets of genotypes) of 1000, 2000 and 3000 were considered for each genotype distribution (for a total of 4×3 = 12 sample size/genotype distribution combinations). Simulated genotypes (representing individuals) were randomly assigned as cases or controls (in equal proportion), in line with the null hypothesis that the locus is not related to the dichotomous disease phenotype.

### Simulating Differential Genotype Errors

Because the focus of our analysis is on rare variants, we only considered genotyping errors between the common homozygote and the heterozygote. Let *ε_01_* = the probability that an individual who is actually the common homozygote is misclassified as the heterozygote, and let *ε_10_* = the probability that an individual who is actually the heterozygote is misclassified as the common homozygote. We considered three types of error models in the main simulation, with additional settings considered in an additional simulation (see *Additional simulation settings*). The three main error models considered were (a) Homozygote to heterozygote errors only (*ε_01_ = ε* and *ε_10_* = 0), (b) Heterozygote to Homozygote errors only (*ε_01_ = 0* and *ε_10_* = *ε*) and (c) Both errors present (*ε_01_* = *ε_10_* = *ε*). We considered three different values for *ε* in the controls: 0.1%, 1% and 5%. To simulate differential errors, the value of *ε* in the cases was then increased to *ε+*0.1%, *ε+*0.5% or *ε+*1%. Thus, in total, the main simulation analysis considers 324 total settings: all possible combinations of the 6 factors (# SNPs (8 or 64), MAF (0.1%/1% or 0.5%/5%, sample size (1000, 2000 or 3000), magnitude of errors (0.1%, 1% or 5%), error model (*ε_01_* only, *ε_10_* only or both *ε_10_* and *ε_01_*) and differential effect in cases (0.1%, 0.5% or 1%)).

### Additional Simulation Settings

Recognizing that because of the nature of genotype calling algorithms, it is likely that the heterozygote to homozygote error rate (*ε_10_*) may be much larger than the homozygote to heterozygote error rate *(ε_01_)* we conducted a small additional simulation study considering error rates reflecting this. Specifically, as in Powers et al. [27], we let *ε_10_ = 10ε_01_*. For this small simulation study, we consider only a sample size of 2000 individuals (1000 cases and 1000 controls), either 8 or 32 SNVs where ¾ of the SNV’s have MAF = 0.001, and ¼ have MAF = 0.01. Furthermore, we investigated three different combinations of genotyping error rates in the controls: 0.1%/1% (*ε_01_/ε_10_*), 1%/10% and 5%/50%. To introduce differential error we increased each error rate, considering four different options: *ε_01_+*0.1% and *ε_10_+*0.1%, *ε_01_+*0.3% and *ε_10_+*0.3%, *ε_01_+*0.5% and *ε_10_+*0.5%, and *ε_01_+*1% and *ε_10_+*1% in the cases. Thus, we considered a total of 24 settings; all possible combinations of number of SNVs (8 or 32), control genotype error rate (0.001/0.01, 0.01/0.10, 0.05/0.5), and differential case error rate (magnitude of change 0.1%, 0.3%, 0.5% or 1%).

### Rare Variant Tests Used to Analyze Data

This paper examines the effects of differential genotyping error through consideration of five commonly used rare variant tests of association: Combined Multivariate and Collapsing (CMC) [1], Weighted-Sum (WS) [2], Proportion Regression (PR) [5], Cumulative Minor Allele Test (CMAT) [7], and Sequence Kernel Association Test (SKAT) [11]. These methods are all described in detail in the original papers proposing these methods, with our specific implementations of the first four methods described in Powers et al. [27]. In the following sections we briefly describe each method, and explain our implementation of SKAT.

### CMC

The combined multivariate and collapsing (CMC) test aggregates mutations at rare variant sites within a defined region according a threshold defined *a priori* (here, all sites were aggregated since we only consider rare variation; MAF<5%). Each individual is assigned a dichotomous variable representing their status across all sites at the locus: a 0 if all sites are wildtype and a 1 if one or more rare variants are present in the region. The asymptotic distribution of Hotelling’s T^2^ is used to evaluate statistical significance.

### WS

The weighted sum (WS) method employs a weighting scheme to increase the signal of the rarest variants. A weight is calculated for each variant site by estimating the standard deviation of the total number of mutations in controls. Each individual is assigned a score that is the sum of the number of minor alleles divided by the weight at each site. Individuals are ranked according to their score. The test statistic is the sum of the rankings of case individuals. Statistical significance is assessed using a permutation approach with 1000 permutations of case/control status.

### PR

In proportion regression (PR), disease status is regressed on a single covariate representing the percentage of sites at the locus which possess a rare variant for the individual. We used logistic regression and the asymptotic distribution of the likelihood ratio statistic to assess statistical significance.

### CMAT

The cumulative minor allele test (CMAT) counts the total number of rare/common alleles within the locus of interest for both cases and controls and uses a test statistic similar in spirit to a Pearson chi-squared test, where permutation is used to assess statistical significance since individuals can contribute multiple counts.

### SKAT

The sequence kernel association test (SKAT) uses a multiple regression model to directly regress the phenotype on genetic variants at the locus. SKAT analyzes the regression coefficients of the variants using a variance component score test using an unweighted linear kernel. P-values are computed analytically. We used the R package provided by the authors of SKAT to generate p-values for our analysis.

### Computing Type I Error

1000 separate simulations of each of the 324 parameter settings in the main simulation study, along with 1000 simulations of the each of the 24 settings in the additional simulation analysis, were used to assess the type I error rate. The type I error rate was assessed as the proportion of the 1000 simulations generating p-values less than 0.05.

## Results

### Overall Assessment of Type I Error Rate Inflation

Across the 324 settings and five rare variant tests, the estimated type I error rate ranged from 1.2% to 100%. However, for each of the five tests, the majority of simulation settings showed meaningfully increased type I error, which we define as a type I error rate above 6.1% (a value which should occur less than 5% of the time by random chance if the true type I error rate is actually 5%). Specifically, for CMC, 62.7% of the 324 settings showed a type I error rate above 6.1%, with similar values for the other four tests (WS 67.6%, PR 69.8%, CMAT 77.5%, SKAT 56.2%). Simulations with no genotype errors but other simulation parameters related to those considered in this manuscript, show either control or slight conservatism in the empirical nominal type I error rate for CMC, WS, PR, and CMAT (Powers et al. 2011). SKAT shows similar patterns (detailed results not shown).

### Impact of Different Factors on Type I Error Rate

In order to understand, generally, how the type I error rate is affected by each of the six simulation parameters, we fit five separate multiple regression models: one for each rare variant test. The model predicted the observed type I error rate by each of the six simulation parameters (where we used relative amount of differential expression, case error rate divided control error rate, instead of magnitude of differential error). Model r^2^ values ranged from 41 to 56% suggesting that the main effects of the six simulation parameters explained the approximately half of the total change observed in the type I error rate.

Table 1 gives the seven coefficients corresponding to the six simulation parameters (there two coefficients for the error model parameter since we use indicator variables) for each of the five rare variant tests. Five of the seven coefficients are significant in each model, suggesting that most simulation factors directly impact the type I error rate. We provide a brief interpretation of the coefficients from Table 1 here. The impact of sample size was positive for all tests, meaning that as sample size increases, type I error rate increases for all tests. Across the settings considered here, type I error increased by between 3.7 to 6.3% for each additional 1000 individuals in the study.

**Table 1:** Coefficients from regression models predicting type I error rate.

Parameter		CMC	WS	PR	CMAT	SKAT
Sample Size		5.6×10^−5^ ***	3.7×10^−5^ *	4.1×10^−5^ **	4.3×10^−5^ **	6.3×10^−5^ ***
Number of variants		1.4×10^−3^ **	3.2×10^−3^ ***	3.4×10^−3^ ***	3.1×10^−3^ ***	2.9×10^−3^ ***
MAF		−9.9×10^−2^ ***	−7.9×10^−2^ **	−8.3×10^−2^ **	−9.5×10^−2^ ***	−1.2×10^−1^ ***
Relative amount of differential error		3.1×10^−2^ ***	2.5×10^−2^ ***	2.9×10^−2^ ***	2.7×10^−2^ ***	3.7×10^−2^ ***
Error rate in controls		−1.9*	6.2	7.9	7.6	1.1
Error model	*ε_01_* only	−1.6×10^−2^	−1.8×10^−2^	−1.8×10^−2^	−1.7×10^−2^	−7.0×10^−3^
	*ε_10_* only	−0.51***	−0.58***	−0.58***	−0.58***	−0.42***

* p<0.05,

** p<0.01,

*** p<0.001.

The Type I error rate also increased as the number of variants increased for all tests. Specifically, for each additional variant added to the test (for which the same differential error model is true), the type I error rate increased between 0.14 and 0.34%.

The impact on type I error rate lessened as the minor allele frequency increased. Specifically, for every 1% increase in minor allele frequency the type I error rate decreased by approximately 7.9 to 12.0%.

As expected, the relative amount of differential error is strongly associated with the observed type I error rate. Namely, as the relative amount of differential error increased, the type I error rate increased. For example, if the relative amount of genotype error (case error rate/control error rate) increases from 2 to 3 (e.g., goes from 2%/1% to 3%/1%), the type I error rate increases by approximately 2.5 and 3.7%. Because we used the relative differential error in our model, the control error rate showed little effect on type I error rate.

The error model coefficients are based on indicator variables. The coefficient for the *ε_10_* only model shows that there is a smaller increase in type I error rate when only *ε_10_* errors occur than when both *ε_10_* and *ε_01_* errors occur. Additionally, there is not a significant difference between the *ε_01_* only error model and the both errors model. The difference in effect is estimated to be between 42% and 63% more type I errors in either model that contains homozygote to heterozygote errors, as compared to a model with only heterozygote to homozygote errors.

**Figure 1:**
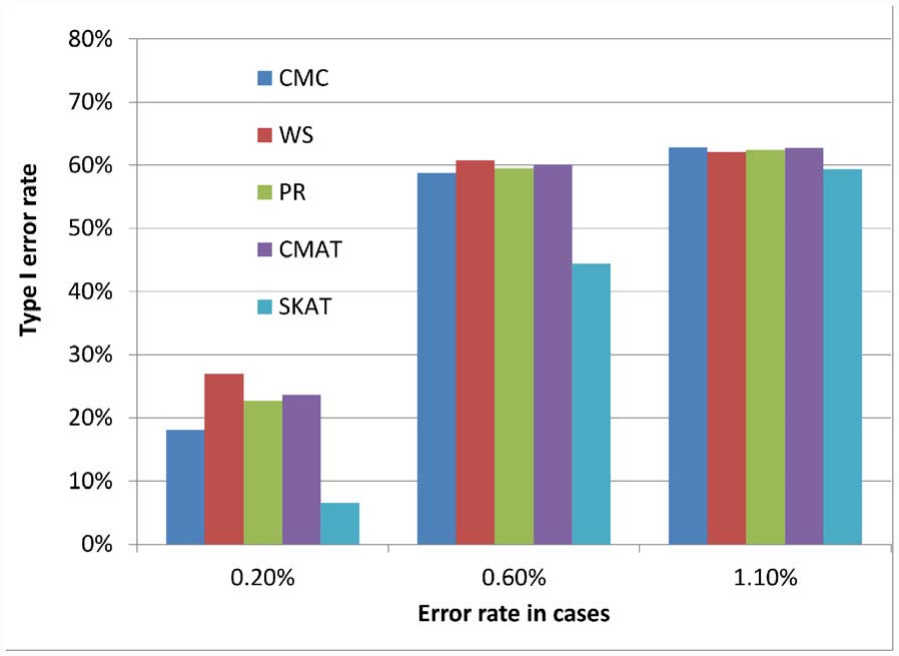
Type I error rate when error rate in controls is 0.10%. The observed type I error rate, averaged across all simulation settings, for each of the five rare variant tests (CMC, WS, PR, CMAT and SKAT). Differential genotyping error can be substantial, even at low error rates.

**Figure 2:**
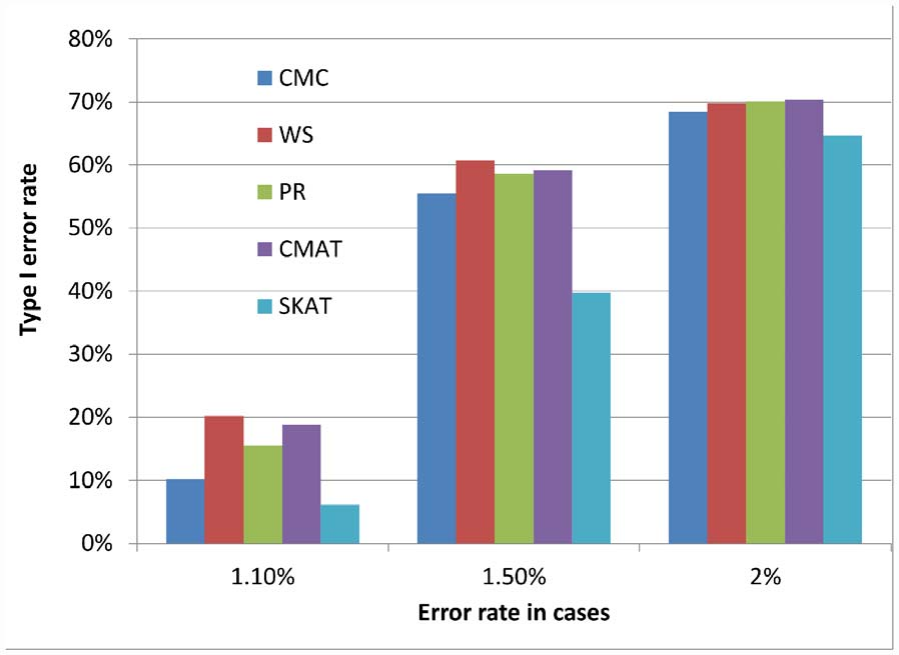
Type I error rate when error rate in controls is 1%. The observed type I error rate, averaged across all simulation settings, for each of the five rare variant tests (CMC, WS, PR, CMAT and SKAT). Modest levels of differential genotyping error rates can substantially increase the type I error rate.

**Figure 3:**
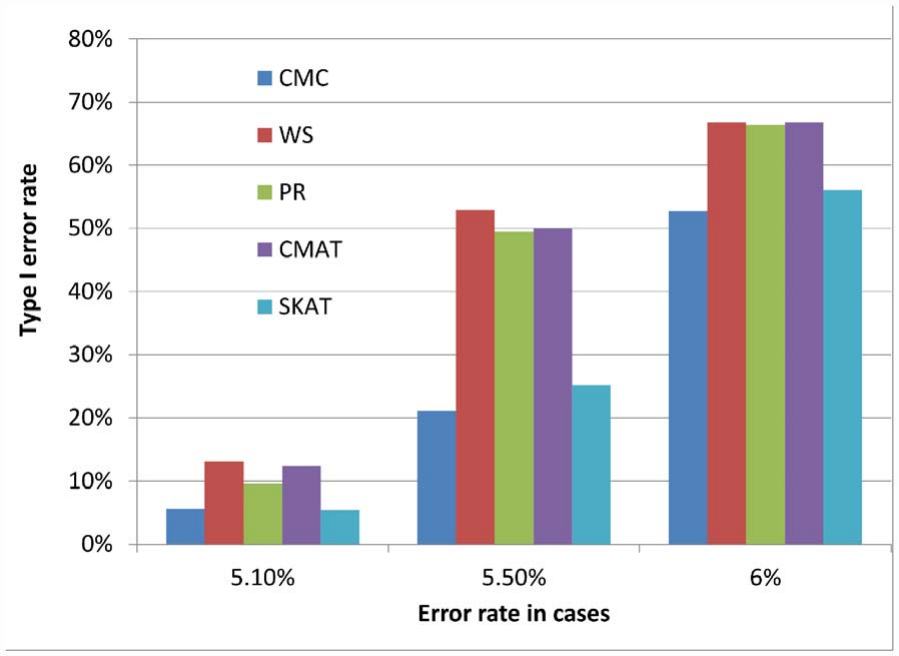
Type I error rate when error rate in controls is 5%. The observed type I error rate, averaged across all simulation settings, for each of the five rare variant tests (CMC, WS, PR, CMAT and SKAT). High levels of differential genotyping errors can substantially increase the type I error rate.

Figures 1, 2, and 3 illustrate the observed type I error rate (averaged across all simulation settings) for each of the five tests, and for each of the nine combinations of case and control error rates. Differential genotyping error, even in low amounts (0.2%) can substantially increase the type I error rate for all five tests.

**Figure 4:**
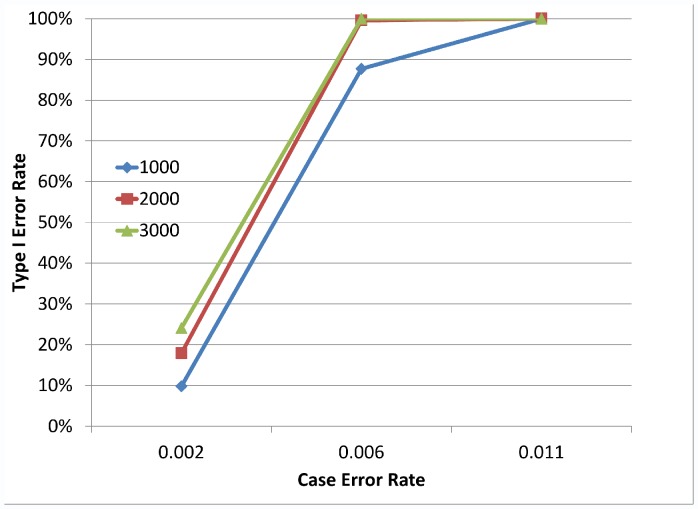
Type I error rate by case genotyping error rate and sample size. An example of how the Type I error rate changes by sample size and amount of differential genotyping error. Notably, as the amount of differential genotyping error increases, and as the sample size increases, the Type I error rate increases. Here we show results from the PR test with a control genotype error rate of 0.1%, ε_01_ = ε_10_, 8 SNVs, with 6 SNVs at MAF = 0.1% and 2 SNVs at MAF = 1%. Different values for the case error rate vary along the x-axis.

As suggested by the multiple regression models, there is a significant impact of sample size on the type I error rate of the different tests. Figure 4 illustrates how the magnitude of the differential errors and the sample size combine to impact the type I error rate. Similar graphs are obtained for all five rare variant tests and other simulation settings (details not shown).

### Analysis of Additional Simulation Settings

**Figure 5:**
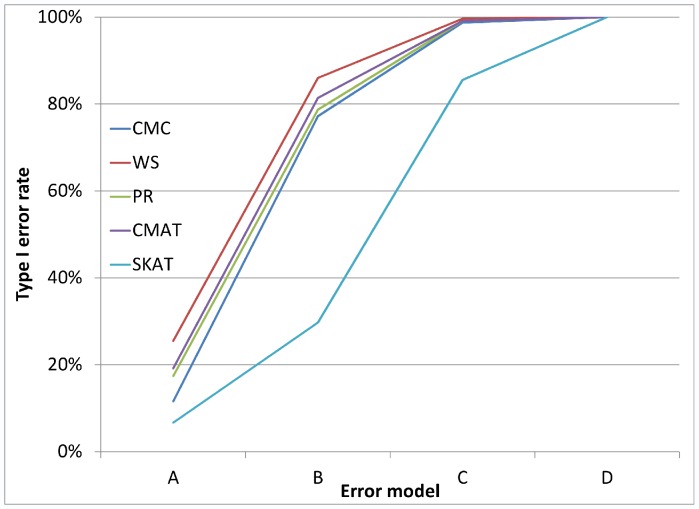
Type I error rate variability by error model for a gene with 8 SNVs. Figure 5 considers a gene containing 8 rare variants. All error models have control error rates fixed at ε_10_ = 1% and ε_01_ = 0.1%. For error model A cases: ε_10_ = 1.1%, ε_01_ = 0.2%, error model B is cases: ε_10_ = 1.3%, ε_01_ = 0.4%, error model C is cases: ε_10_ = 1.5%, ε_01_ = 0.6% and error model D is cases: ε_10_ = 2.0%, ε_01_ = 1.1%. Type I error increases for all error models as the genotyping error rate increases.

**Figure 6:**
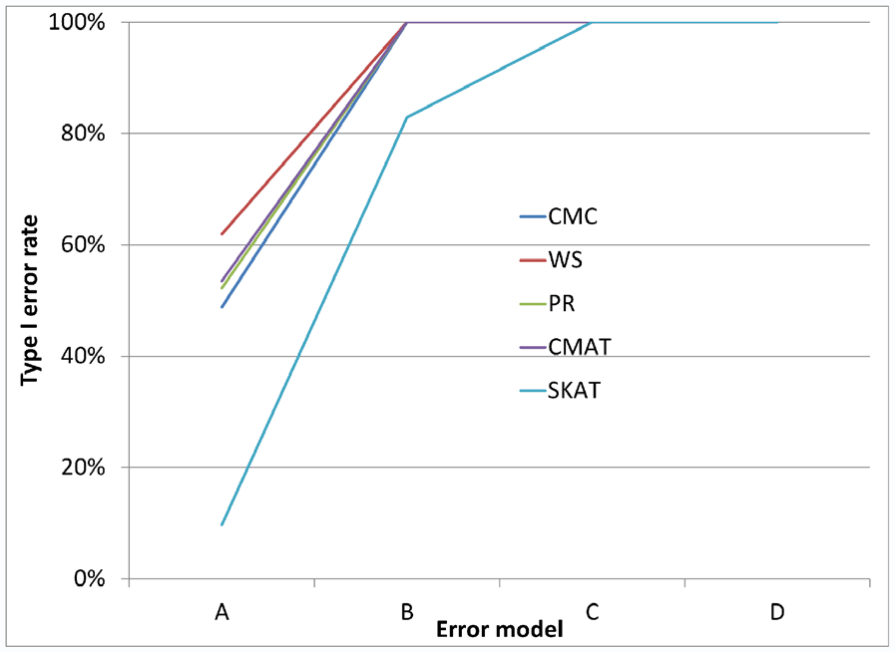
Type I error rate variability by error model for a gene with 32 SNVs. Figure 6 considers a gene containing 32 rare variants and considers the same error models as are in Figure 5.

**Figure 7:**
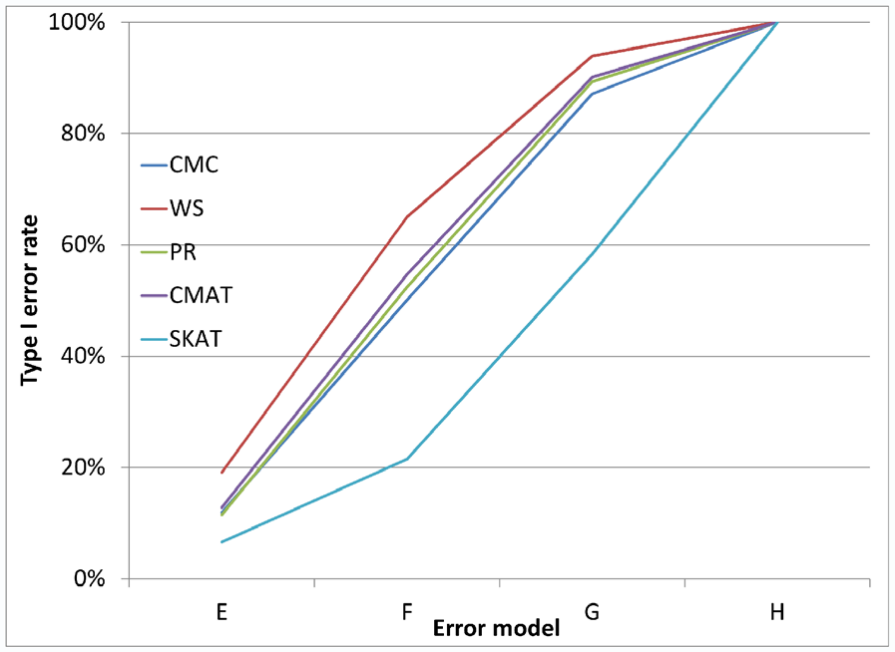
Type I error rate variability across additional error models: a gene with 8 SNVs. Figure 7 considers loci with 8 rare variants. All error models have controls: ε_10_ = 10% and ε_01_ = 1%. For error model E cases: ε_10_ = 10.1%, ε_01_ = 1.1%, error model F is cases: ε_10_ = 10.3%, ε_01_ = 1.3%, error model G is cases: ε_10_ = 10.5%, ε_01_ = 1.5% and error model H is cases: ε_10_ = 11.0%, ε_01_ = 2%.

**Figure 8:**
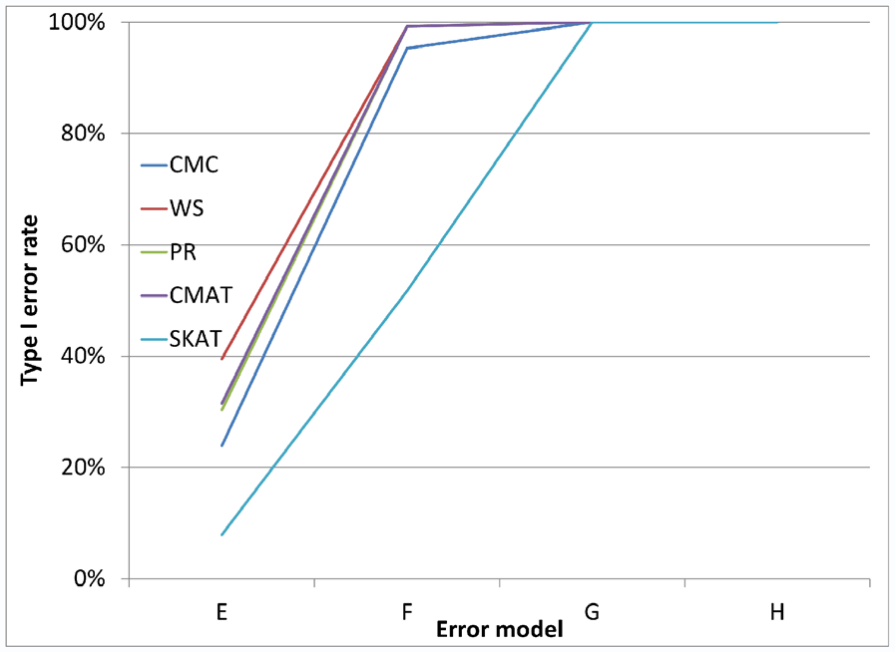
Type I error rate variability across additional error models: a gene with 32 SNVs. Figure 8 considers loci with 32 rare variants and considers the same error models as are in Figure 7.

**Figure 9:**
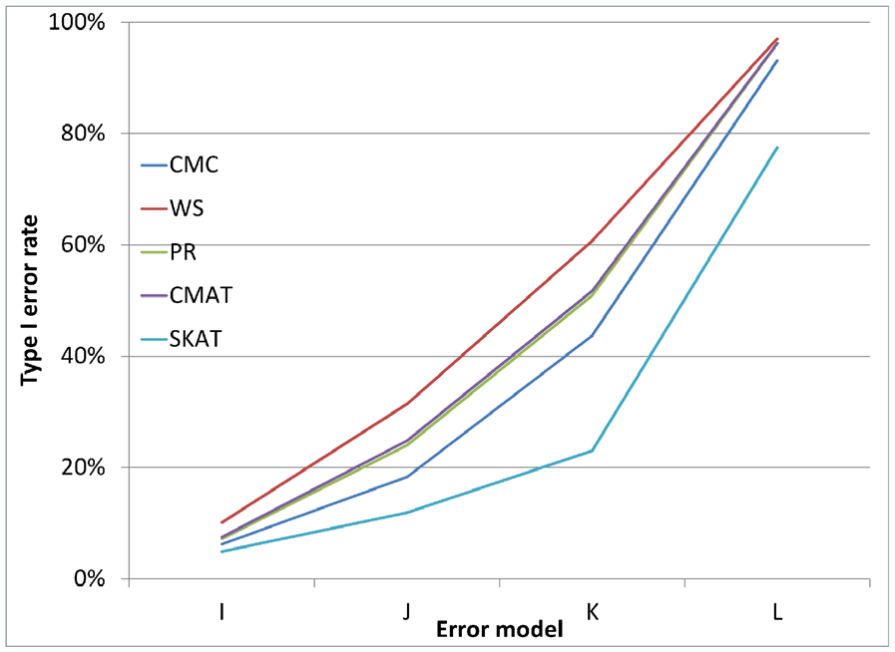
Type I error rate variability across additional error models: a gene with 8 SNVs. Figure 9 considers loci with 8 rare variants. All error models have controls: ε_10_ = 50% and ε_01_ = 5%. For error model I cases: ε_10_ = 50.1%, ε_01_ = 5.1%, error model J is cases: ε_10_ = 50.3%, ε_01_ = 5.3%, error model K is cases: ε_10_ = 50.5%, ε_01_ = 5.5% and error model L is cases: ε_10_ = 51.0%, ε_01_ = 6%.

**Figure 10:**
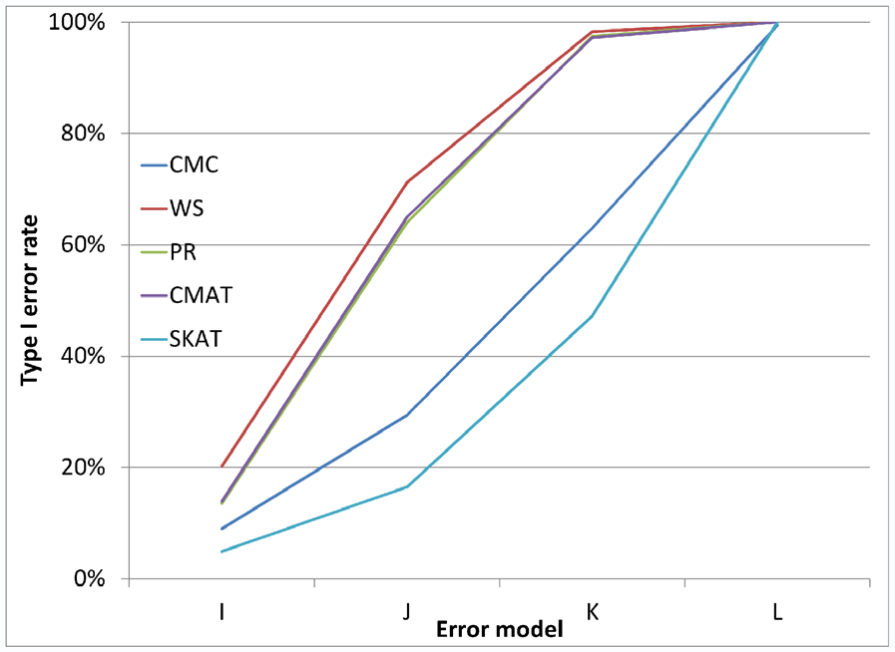
Type I error rate variability across additional error models: a gene with 32 SNVs. Figure 10 considers loci with 32 rare variants and considers the same error models as are in Figure 9.

As noted earlier, in addition to the main simulation study which covered 324 settings, we also conducted a small simulation study covering 24 settings, which were selected to reflect genotyping error patterns that may be observed based on current genotype calling algorithms. Figures 5 and 6 each illustrate the type I error rate for four of the twenty-four settings. In each case, we see that even for very small amounts of differential genotyping error, substantially inflated type I errors can be observed. Additional figures showing similar patterns for differential error models with higher error rates show similar patterns (see Figures 7, 8, 9, and 10 for details).

## Discussion

Our results demonstrate the potential for inflation of the type I error rate in a variety of error models, across five commonly used rare variant tests of association. Furthermore, as has been demonstrated for single marker tests of association, increasing the sample size and decreasing the minor allele frequency both inflate the type I error rate. Additionally, since rare variant tests aggregate across multiple sites, increasing the number of SNVs for which differential genotyping error is present increases the type I error. Lastly, as has been demonstrated for single marker tests, differential errors which increase homozygote to heterozygote genotyping errors are particularly detrimental.

Our results also demonstrate that increasing the relative amount of the differential error inflates the type I error rate, while we did not find strong evidence of an effect of the control genotyping error rate. While we only considered three settings for the control error rate, these results are interesting because they suggest that even low amounts of genotyping error can be problematic if the relative differential genotyping error is large (e.g., low error rates of 0.1% in controls and 1.0% in cases, but large relative differential error value of 10 = 1%/0.1%).

Intuitively, type I errors are introduced from differential genotyping errors, because, under the null hypothesis of no association between the locus and the phenotype, the allele frequency distributions are identical. Non-differential genotyping errors do not increase the type I error rate, because the allele frequency distributions remain the same for cases and controls. However, differential genotyping errors introduce a difference between the case-control allele frequencies, in the same manner as if there were a true genotype-phenotype association at the locus. Because differential genotype errors manifest themselves as different allele frequency distributions between cases and controls, all of the variables that impact the power of a rare variant test of association will also impact the type I error rate in the presence of differential genotyping error. Specifically, increasing the sample size will increase the power for testing a true effect, just as it increases the type I error in the presence of differential genotyping errors. Similarly, the relative amount of differential genotyping errors, the number of SNVs at the locus, the MAF and the type of errors observed (homozygote to heterozygote or vice versa) are all related to type I errors.

This intuition helps to not only explain why certain factors are related to the type I error rate, but also points to areas of concern and caution for researchers today. For example, increasingly researchers are turning to meta-analytic techniques and combining datasets across multiple labs, or using publicly available controls. The goal, of course, is to increase power by increasing sample size. A significant concern raised by our analysis is that if different error processes are present in the samples in a manner associated with the phenotype, there is the potential for significant type I error problems.

As noted in the text, there were also numerous settings considered in our analyses where the type I error rate did not inflate. In general, these settings were when the sample size was small, the MAF was large and the differential genotyping errors impacted the heterozygote to homozygote genotyping errors. While important to note that type I errors will not always inflate in the presence of differential genotyping errors, power will also tend to be low in many such studies. Essentially, as the overall power of a study increases, so does the potential that the study is impacted by an inflated type I error rate.

Despite the rush to justify the existence of next-generation sequencing data, caution and attention to quality study design techniques will be critical. This means researchers must ensure random assignment of cases and controls to sequencing locations and using basic quality control procedures on sequencing data. For example, Q–Q plots are commonly used to detect large-scale type I error problems (e.g., from population stratification), and thus are a practical way to detect data-set wide type I error problems. Of course, individual loci that may be affected by type I errors will not be identified by this approach, which underscores that careful design strategies still must be employed, and replication of significant findings is necessary. Furthermore, there are a host general quality control steps that should be taken in an NGS study. While best-practices continue to develop, evaluating data quality (e.g., individual SNP quality via BAM files) and designing studies to include some redundancy (e.g., technical replicates and/or genotyping some participants with arrays) are generally recommended and may help to identify genotype errors before they impact downstream statistical analyses.

Importantly, we note that our findings apply not only to next-generation sequencing technology, but to imputed variants or variants genotyped on an array as well. Our approach is not technology specific, but considers the impact of differential errors on the statistical methods used to analyze rare variant data, regardless of the technology used to generate the variant calls.

There are some limitations of our analysis worth noting. We use simulated genotype data which ignores LD, and simplifies the true allele frequency distribution observed in next-generation sequencing data. These simplifying assumptions are common in methodological papers on rare-variant tests of association proposed to date, however further analysis is necessary to project these findings to more realistic sequence data. Second, the goal of this paper was not to compare which rare variant tests may be more or less resistant to certain types of genotyping errors. Notably, in our analyses SKAT appear to be more resistant to type I errors than the other methods. However, SKAT was designed to perform optimally in situations where there is a mix of signals, resulting from a combination of neutral, protective and risk variants and has been observed to have a conservative type I error rate in some settings. Recent work (Liu, unpublished manuscript) also provides a general framework for rare variant tests which classifies CMC, PR, CMAT and WS differently than SKAT, which may also explain some of the differences observed here. Additionally, more complex error models should be considered as more is learned about the error processes involved next-generation sequencing data. Fourth, our analysis of type I errors considers a significance level of 5%, which is unrealistic for large genome-wide studies. Without analytic consideration of the different tests or substantially more computation time to simulate data, estimates of type I error rates at lower significance levels cannot be obtained. However, as has been found with single marker tests, we have no reason to believe that the patterns of results will be different at different significance levels. Finally, our choice to use only additive main effects in the multiple regression model is a simplistic one that does not completely reflect the underlying complexity (non-linear relationships; interactions, etc.) of the relationship between the six parameters considered here and type I error. A more detailed analysis should be conducted for any particular study design or error pattern of interest. However, given that the models explained a significant portion of the variance in the type I error rate, they can be interpreted as giving a general sense of the true relationships.

Our analysis has demonstrated that type I errors caused by differential genotyping errors could be a significant problem in rare variant tests of association applied to next-generation sequencing data. In fact, some early application of the tests to real sequencing data suggests this could be the case. Careful consideration of study design, caution in meta-analysis and using publicly available controls, and use of standard quality control metrics is critical in an effort to minimize type I errors. Further work is necessary to fully characterize and explore the creation and consequences of differential genotyping errors in rare variant tests of association.
